# Effect of fenfluramine on seizures and comorbidities in *SCN8A*‐developmental and epileptic encephalopathy: A case series

**DOI:** 10.1002/epi4.12623

**Published:** 2022-07-20

**Authors:** Ángel Aledo‐Serrano, Borja Cabal‐Paz, Elena Gardella, Pablo Gómez‐Porro, Otilia Martínez‐Múgica, Alvaro Beltrán‐Corbellini, Rafael Toledano, Irene García‐Morales, Antonio Gil‐Nagel

**Affiliations:** ^1^ Neurology Department, Epilepsy Program Ruber Internacional Hospital Madrid Spain; ^2^ Neurology Department Puerta de Hierro University Hospital Madrid Spain; ^3^ Department of Epilepsy Genetics and Personalized Treatment, Member of ERN‐Epicare Danish Epilepsy Center Dianalund Denmark; ^4^ University of Southern Denmark Odense Denmark; ^5^ Pediatric Neurology Section, Pediatrics Department Donostia University Hospital San Sebastian Spain; ^6^ Epilepsy Unit, Neurology Department Ramon y Cajal University Hospital Madrid Spain; ^7^ Epilepsy Unit, Neurology Department Clínico San Carlos University Hospital Madrid Spain

**Keywords:** drug repurposing, epilepsy, genetic epilepsy, seizures, sodium channelopathy

## Abstract

*SCN8A*‐developmental and epileptic encephalopathy is caused by pathogenic variants in the *SCN8A* gene encoding the Na_v_1.6 sodium channel, and is characterized by intractable multivariate seizures and developmental regression. Fenfluramine is a repurposed drug with proven antiseizure efficacy in Dravet syndrome and Lennox–Gastaut syndrome. The effect of fenfluramine treatment was assessed in a retrospective series of three patients with intractable *SCN8A* epilepsy and severe neurodevelopmental comorbidity (n = 2 females; age 2.8–13 years; 8–16 prior failed antiseizure medications [ASM]; treatment duration: 0.75–4.2 years). In the 6 months prior to receiving fenfluramine, patients experienced multiple seizure types, including generalized tonic–clonic, focal and myoclonic seizures, and status epilepticus. Overall seizure reduction was 60%–90% in the last 3, 6, and 12 months of fenfluramine treatment. Clinically meaningful improvement was noted in ≥1 non‐seizure comorbidity per patient after fenfluramine, as assessed by physician‐ratings of ≥“Much Improved” on the Clinical Global Impression of Improvement scale. Improvements included ambulation in a previously non‐ambulant patient and better attention, sleep, and language. One patient showed mild irritability which resolved; no other treatment‐related adverse events were reported. There were no reports of valvular heart disease or pulmonary arterial hypertension. Fenfluramine may be a promising ASM for randomized clinical trials in *SCN8A*‐related disorders.

## 1 INTRODUCTION

*SCN8A*‐related disorders result from pathogenic variants in the gene on chromosome 12q13.13 that encodes the alpha subunit of the Na_v_1.6 sodium channel.^1^, ^2^ The phenotypes associated with *SCN8A* pathogenic variants range in severity—from benign to severe developmental and epileptic encephalopathies (DEEs) with seizures of multiple types.^2^ Patients experience developmental delay, intellectual impairment, abnormal neurodevelopment, movement disorders, language impairment, and sleep problems.^1^ Patients with *SCN8A* epilepsies are at an elevated risk of sudden unexpected death in epilepsy (SUDEP); SUDEP was reported in ~10% of patients with *SCN8A‐*related disorders in a recent systematic review of 56 studies (N = 235 patients).^3^ Seizures and comorbidities are persistent and mostly treatment‐resistant, despite polypharmacy.^3^, ^4^, ^5^, ^6^, ^7^

Studies with the repurposed drug and antiseizure medication (ASM) fenfluramine—a potent serotonin releaser with positive modulatory activity at Sigma‐1 receptors and agonist activity at 5‐HT receptors (5‐HT_2_, with additional evidence for 5‐HT_1D_ and 5‐HT_4_)^8^, ^9^—have demonstrated decreased seizure frequency in patients with DEEs, including Dravet syndrome (DS), Lennox–Gastaut syndrome (LGS), Sunflower syndrome, and *CDKL* deficiency disease (CDD).^8^, ^10^, ^11^, ^12^ In randomized clinical trials (RCT) and open‐label extension studies (OLEs), fenfluramine was effective and well‐tolerated, with no observations of pulmonary arterial hypertension or valvular heart disease.^8^, ^10^, ^11^, ^12^

Here, we present a case series of three patients with *SCN8A*‐related DEE treated with fenfluramine.

## 2 METHODS

Patients in this retrospective case series were being treated at Ruber Internacional Hospital (Madrid, Spain; n = 2) or Donostia University Hospital (San Sebastián, Spain; n = 1). Fenfluramine treatment was initiated by compassionate use in the European Early Access Program or in one of the phase 3 trials. Data collection and analysis were approved by the ethics committees in Ruber Internacional Hospital. Informed consent was obtained for every patient through their caregivers. Patients were treated with fenfluramine at a starting dose of 0.2 mg/kg/day and titrated to effect. At analysis, monthly seizure frequency during the 6 months prior to fenfluramine treatment was evaluated. Comorbidities were evaluated qualitatively and with investigator ratings on the Clinical Global Impression of Improvement (CGI‐I) scale, a 7‐point Likert scale. Echocardiograms were performed at baseline before treatment with fenfluramine and then every 3–6 months after fenfluramine therapy was initiated.

## 3 RESULTS

The three patients (two females and one male) had genetically confirmed *SCN8A* pathogenic variants—one deletion/frameshift mutation (Patient 1) and two missense point mutations (Patients 2 and 3, with mosaicism in patient 2; Table 1). Disease durations were 3, 7, and 12 years, respectively. Patients had a treatment‐resistant epilepsy, with a history of eight to 16 prior failed ASMs. The births of all three patients were uncomplicated; they were developmentally normal or showed mild delay until seizure onset; Patient 1 developed ventricular dilatation shortly after birth. All patients exhibited developmental delay after seizure onset, with an intellectual disability rating of “severe/very severe.” Patient 1 (age 7.5 years at fenfluramine treatment initiation) developed only syllabic language skills with regression by age 3 years, and had involution of visual connection (cortical blindness); his motor impairment was evident in ambulation delay until 4 years, with gait instability and truncal ataxia. Patient 2 (age 13 years) showed neurodevelopmental slowing after 12 months. By 24 months she was irritable, impulsive, and inattentive. By 24 months, she could speak in brief sentences and became ambulant, but by age 4 years, she had regressed to non‐verbal and non‐ambulant, and she required full assistance for all activities and transfers. Patient 3 (age 2.6 years) showed neurodevelopmental impairment; she did not achieve new developmental milestones; and she experienced regression and loss of cephalic support. Patient 1 harbored a frame shift pathogenic variant suggesting a possible loss‐of‐function (LOF) effect. Meanwhile, Patients 2 and 3 showed a clinical picture typical for gain‐of‐function (GOF). Indeed, they harbored pathogenic variants predicted to have a GOF effect, although response to sodium channel blockers was heterogeneous.

**TABLE 1:** Patient characteristics

#	Sex	*SCN8A* Pathogenic Variant	vEEG	MRI	Seizure Types		Comorbidities	ASMs		Age (years)			FFA Treatment Duration		
					Prior	BL		Prior (n)^a^	At FFA Start^c^	At Epilepsy Onset (mo)	At 6 mo Before FFA Start (yr)	At FFA Start (yr)	Time to Target Dose (days)	Total Duration (yr)	Target dose (mg/kg/day)
1	M	c.324del p.Thr109fs *SCN8A*	Background slowing at bilateral parieto‐occipital region Spike‐wave complexes in the left parieto‐occipital region with contralateral propagation	Corpus callosum hypoplasia Mild cerebral atrophy	TS (hypertonic, ocular) FIA HC (left)	GTC EM	Neurodevelopmental delay, irritability, behavioral problems. Non‐verbal	16^b^	BRV CLB ZNS (*added during FFA*)	1	7.0	7.5	120	3.7	0.7
2	F	c.2629G>A; p.Ala874Thr *SCN8A* (mosaic 19.2%)	Background slowing and bilateral frontal epileptiform activity, with right hemisphere predominance	Normal	BCS Convulsive SE TS (bilateral, predominantly left) FS (complex)	GTC FS	Neurodevelopmental delay after 12 months with irritability, impulsivity, inattention. Non‐ambulant and non‐verbal by 4 years old.	8^b^	ZNS (*dose incr during FFA*)	9	12.5	13	360	4.2	0.7
3	F	c.2620G>A p.Ala874Thr *SCN8A*	*Before FFA:* Spasms, multifocal activity at age 8 mo Worsening with reappearance of hypsarrhythmia at age 10‐14 mo Frequent multifocal and polymorphic epileptiform activity at age 14 mo *After FFA:* Mild background slowing, without epileptiform activity	Transient abnormalities in brainstem, superior cerebellar peduncles, and basal ganglia, possibly related to vigabatrin at first year of life	‐‐	GTC ES FS (nocturnal) TS (sporadic)	Neurodevelopmental delay; did not achieve developmental milestones; regression and loss of cephalic support	12^b^	CZP OXC TPA VGB ZNS	4	2.6	3.1	45	0.75	0.7
Median (range)			‐‐	‐‐	‐‐	2 (2‐4)	‐‐	12 (8‐16)	3 (1‐5)	4 (1‐9)	7 (2.6‐12.5)	7.5 (3.1‐13)	120 (45‐360)	3.7 (0.75‐4.2)	0.7

Abbreviations: ASM, antiseizure medication; BL, baseline 6 mo before FFA treatment; BRV, brivaracetam; CLB, clobazam; CZP, clonazepam; DEE, developmental and epileptic encephalopathy; FFA, fenfluramine; HC, hemiconvulsion; OXC, oxcarbazepine; vEEG, video electroencephalogram; VGB, vigabatrin; ZNS, zonisamide. Seizure types: BCS, bilateral clonic seizures; ES, epileptic spasm; FIA, focal with impaired awareness; FS, focal seizures; GTC, generalized tonic–clonic; EM, eyelid myoclonia; SE, status epilepticus; TS, tonic seizure.

^a^ Patients 1 and 2 were on a ketogenic diet as a prior, non‐ASM treatment. Patient 3 was on a ketogenic diet until month 7 of FFA (discontinued due to kidney stones).

^b^ Doses. Patient 1: Brivaracetam (50–25–50 mg; 4.43 mg/kg/day), Clobazam (5 mg/8 hr; 0.53 mg/kg/day); zonisamide added during FFA treatment (0–0–50, then 0–0–75 mg; 1.61 mg/kg/day); Patient 2: Zonisamide (175 mg/day initially; increased to 300 mg/day during FFA treatment); Patient 3: clonazepam 0.04 mg/kg/day at night, oxcarbazepine 20 mg/kg/day (withdrawn during 6 wk' titration period), topiramate (3.13 mg/kg/day; withdrawn during the 6 wk' titration period), vigabatrin 140 mg/kg/day (withdrawn during 6 wk titration period), and zonisamide (100 mg/12 hr, added at Month 7). Patient 3 was also on ketogenic diet at FFA initiation, but terminated diet at Month 7 due to kidney stones.

^c^ Prior ASMs. Patient 1: clonazepam, corticosteroids, eslicarbacepine, ethosuximide, lamotrigine, levetiracetam, oxcarbazepine, perampanel, phenytoin, phenobarbital, primidone, rufinamide, topiramate, vigabatrin; worsening with sodium channel blockers: carbamazepine, lacosamide. Patient 2: cannabidiol, clobazam, levetiracetam, oxcarbazepine, perampanel, topiramate, valproic acid, stiripentol. Patient 3: levetiracetam, vigabatrin, vitamin cocktail [pyridoxine, biotin, folinic acid], valproic acid, vigabatrin, topiramate, oxcarbazepine, clonazepam; phenytoin; intramuscular ACTH.

Abnormalities on MRIs were evident in two patients. Patient 1 showed evidence of corpus callosum hypoplasia with mild cerebral atrophy. Patient 3 showed transient alterations in the brainstem, superior cerebellar peduncles, and basal ganglia 4 months after seizure onset, probably related to vigabatrin treatment. EEGs showed slowing of background activity in all three patients, with evident epileptiform activity prior to fenfluramine treatment (Table 1).

Disease onset occurred between ages 1 and 9 months. Patient 1 presented at 1 month with tonic seizures (hypertonia/ocular version). New seizure episodes were evident at 1 year of age, focal seizures with impaired awareness (occasionally temperature‐sensitive) at 2 years, eyelid myoclonia from 2 to 5 years, and left hemiconvulsion with evolution to status epilepticus at 5 years. In the 6 months prior to fenfluramine initiation, Patient 1's seizures were generalized tonic–clonic (GTCS) (5/month), focal with impaired awareness and desaturation (2–5 times/day, 30–180/month), and to a lesser extent, eyelid myoclonia (90/month); he also had one episode of status epilepticus (6 total, lifetime). Patient 2 presented at 4 months with a 4 minute febrile GTCS. By 12 months, she had two more afebrile bilateral clonic seizures without fever/systemic infection, although she was also recovering from otitis media when the seizures occurred. At 4 years, she developed bilateral tonic activity (predominantly left, during sleep) and an uncountable number of focal seizures. In the 6 months prior to fenfluramine treatment, she was experiencing GTCS (50/month) and focal seizures (15/month). Patient 3 presented at 4 months with GTCS, proceeding to epileptic spasms with multifocal activity on the EEG by 7 months. Seizures persisted at 8 months, with bilateral tonic seizures, and occasional focal seizures in an extremity. In the 6 months prior to fenfluramine (2.6 years), she experienced GTCS (every 2–3 days), daily epileptic spasms, daily focal seizures, and one episode of status epilepticus.

Median age at fenfluramine treatment was 7.5 years (range, 3.1–13), with a median treatment duration of 3.7 years (range, 0.8–4.2 years). Fenfluramine doses began at 0.2 mg/kg/day and were titrated to 0.7 mg/kg/day. At time of analysis, treatment durations at the target dose were 120, 360, and 45 days for Patients 1, 2, and 3, respectively. At fenfluramine treatment initiation, patients had regimens of one to four concomitant ASMs. Vigabatrin, oxcarbazepine, and topiramate were withdrawn from Patient 3's treatment regimen during the 6 week titration period to 0.7 mg/kg/day of fenfluramine. All three patients' regimens included zonisamide at time of analysis. During fenfluramine treatment, Patient 1 had zonisamide added to his regimen and Patient 3 had concomitant zonisamide dose increased; Patient 2's zonisamide dose was stable for the treatment duration.

**FIGURE 1:**
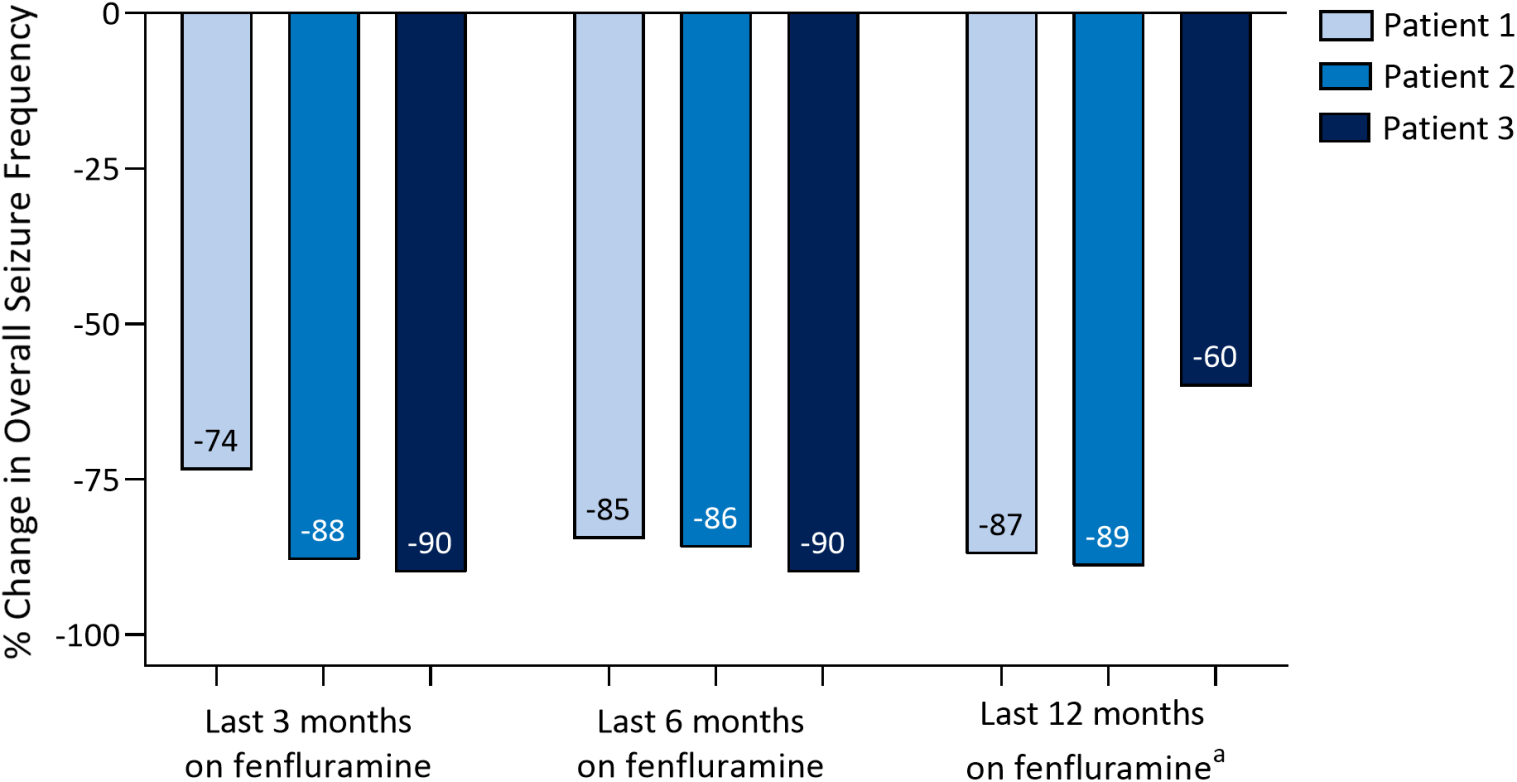
Change in overall seizure frequency during the last 3–12 mo of fenfluramine treatment. ^a^Patient 3 was treated with fenfluramine for 9 mo, including 45 days initial titration and 7.5 mo at the target dose (0.7 mg/kg/day).

All patients achieved a marked reduction in overall seizure frequency after fenfluramine treatment (Figure 1; overall median, −87% change). Patient 1 achieved freedom from GTCS, decrease in frequency in focal seizures after the dose was titrated to 0.7 mg/kg/day fenfluramine, and improvement in seizure intensity. Overall, mean monthly convulsive seizure frequency reduced ≥73% in the last 3, 6, and 12 months of fenfluramine treatment. Patient 2 achieved a decrease in frequency of bilateral focal seizures from 15 to 3 to 6 per month and a decrease in monthly GTCS frequency (≥86% overall) after fenfluramine treatment, without change in seizure severity. Patient 3, after 6 months of fenfluramine but before the addition of zonisamide, achieved a marked decrease in seizure number, with freedom from epileptic spasms and GTCS. Focal nocturnal seizures and sporadic seizures persisted. During fenfluramine treatment, her EEG still showed slowing of background activity, but with absent epileptic activity and reduced quantity of multifocal spikes. After zonisamide was added, Patient 3 achieved a marked improvement in seizures (overall seizure reduction, 60%–90%). Initially, she experienced a period of 20 consecutive seizure‐free days, then she started to have persistent brief focal seizures (seconds in duration) every 5–7 days, as well as sporadic tonic seizures. None of the patients had an episode of status epilepticus after initiating fenfluramine.

**FIGURE 2:**
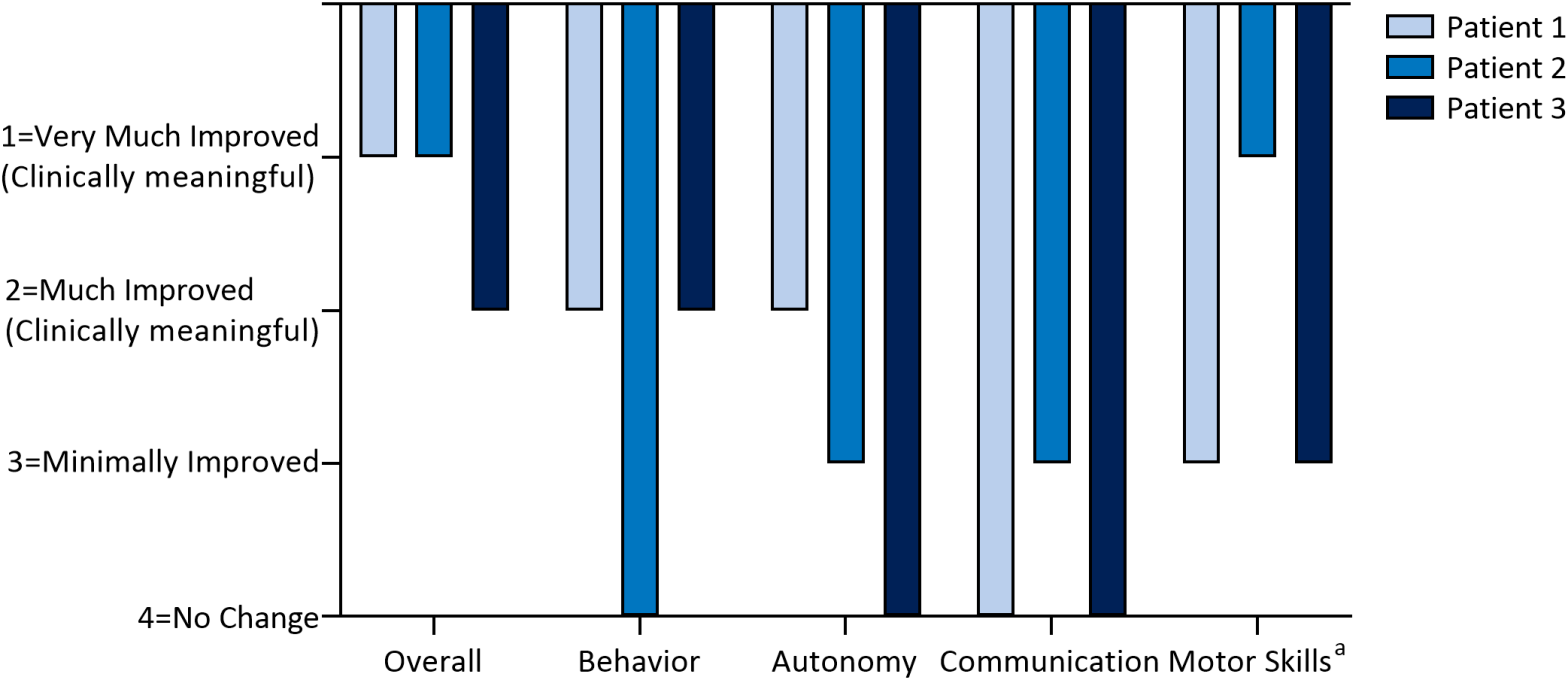
Change in physician‐rated CGI‐I after fenfluramine. No patient was rated as having worsened on the CGI‐I (ratings of 5, 6, or 7). ^a^Patient 2 had regressed to non‐ambulant by 4 y of age (before fenfluramine treatment) and became ambulant after fenfluramine treatment.

Physicians rated all patients as having clinically meaningful improvement in at least one nonseizure comorbidity after fenfluramine treatment (≥“Much Improved” on the CGI‐I; Figure 2). Patient 1 demonstrated greater attention, better sleep, less irritability, and motor improvement. At Month 10 of fenfluramine treatment, he gained the ability to walk down stairs for the first time. Patient 2 was non‐ambulatory and non‐verbal at baseline. After treatment with fenfluramine, she became ambulatory, showed a partial improvement in language, and had improved interaction with her environment. Patient 3 showed clinically meaningful improvement in behavior.

No patient withdrew, discontinued fenfluramine, or experienced a dose‐limiting treatment‐emergent adverse event (TEAE). No serious adverse events were reported. Irritability was noted in Patient 2, which spontaneously resolved over time and was mild in severity. No other TEAEs were reported. None of the patients developed pulmonary arterial hypertension or valvular heart disease.

## 4 DISCUSSION

To our knowledge, this case series is the first report of fenfluramine use to treat patients with genetically‐confirmed *SCN8A*‐related disorder. Fenfluramine was safe and effective in all three patients despite prior treatment resistance (8–16 failed ASMs), particularly when combined with zonisamide. All three patients were responders (≥50% seizure reduction), with a median of 87% reduction in seizures overall in the last 3, 6, and 12 months of fenfluramine treatment. All patients achieved clinically meaningful improvement on at least one non‐seizure comorbidity (scores of 1 or 2, ≥Much Improved) on physician‐rated CGI‐I, including one previously non‐ambulant patient who became ambulant.

The unique mechanism of fenfluramine at both serotonergic and Sigma1R targets^8^ may support fenfluramine as an add‐on therapy option to achieve clinically meaningful, durable reduction in seizure frequency and comorbidity management in patients with *SCN8A*‐epilepsy. In DEEs with a genetic etiology, the underlying pathology contributes—at least in part—to comorbidities independent of seizure burden, as demonstrated in patients with *SCN8A*‐associated disease who are cognitively impaired but do not develop epilepsy.^13^ The preliminary observations in this case series of patients with *SCN8A‐*epilepsy support further investigation into fenfluramine‐mediated improvement in non‐seizure comorbidities. Post‐hoc analyses of phase 3 studies in DS and LGS support improved executive functioning during the relatively short 14 weeks trial duration, and in longer‐term (1 year) OLEs in patients with DS, suggesting durable improvements.^14^ Further investigation is needed to determine whether fenfluramine treatment can slow or alter the natural history of *SCN8A‐*epilepsy as a syndromic condition, including amelioration of the neurodevelopmental delay and regression that lead to psychomotor, behavioral, language, and intellectual impairment.

The potent reduction in GTCS in the patients with *SCN8A*‐DEE in our case series concurs with prior reports.^8^, ^10^ Interestingly, treatment response was homogeneous for three patients with different *SCN8A*‐related disorders: one patient harboring a variant with supposed loss‐of‐function effect (Patient 1) and two patients with likely gain‐of‐function variants, one of them with a mosaicism. The mechanism underlying fenfluramine’s effectiveness in patients with both types of *SCN8A* variants remains to be established, but may be due to enhancement of inhibitory GABAergic input via activity at serotonergic and Sigma‐1 receptors.^8^ In RCTs and OLEs, fenfluramine treatment potently and durably reduced convulsive seizure frequency in patients with DS^8^, ^11^, ^12^ and GTCS frequency in patients with LGS.^8^, ^10^ High burden of GTCS places patients at elevated risk of SUDEP.^15^ Preclinical SUDEP models demonstrated that seizure‐induced respiratory arrest following GTCS was reduced after fenfluramine administration independently of seizure control, and was mediated by serotonin receptor subtypes, including 5‐HT_4_, which is also implicated in learning and memory.^16^ Clinical evidence supports a reduction in SUDEP risk in patients with DS after fenfluramine treatment.^17^ Given that SUDEP has been reported in ~10% of patients with *SCN8A* epilepsy (although the adult phenotype is not well‐documented yet,^3^ and these patients have a high burden of GTCS,^1^, ^3^, ^18^ and typically have ictal respiratory impairment, with O_2_ desaturation with cyanosis^3^) fenfluramine may have important implications for lowering SUDEP risk in patients with *SCN8A*‐epilepsy.

In conclusion, our preliminary results support further investigation and/or RCTs of fenfluramine in treating both seizures and non‐seizure comorbidities in patients with *SCN8A*‐related DEE and potentially other DEEs of genetic etiology, especially syndromes with a high GTCS burden.

## CONFLICTS OF INTEREST

Dr. Aledo‐Serrano received funding for research and educational activities from Zogenix, GW, UCB, Bial, Eisai, Sanofi, Neuraxpharm, and Arvelle. Dr. Cabal‐Paz, Dr. Gardella, Dr. Gómez‐Porro, Dr. Martínez‐Múgica, Dr. Beltrán‐Corbellini, Dr. Toledano, and Dr. García‐Morales have no disclosures. Dr. Gil‐Nagel reports personal fees or research grants from Arvelle Therapeutics, Bial, Biocodex, Eisai, Esteve, GW Pharma, PTC Therapeutics, Sanofi, Stoke, UCB, and Zogenix.

## ETHICAL APPROVAL

We confirm that we have read the Journal’s position on issues involved in ethical publication and affirm that this report is consistent with those guidelines.
